# Heritable clustering and pathway discovery in breast cancer integrating epigenetic and phenotypic data

**DOI:** 10.1186/1471-2105-8-38

**Published:** 2007-02-01

**Authors:** Zailong Wang, Pearlly Yan, Dustin Potter, Charis Eng, Tim H-M Huang, Shili Lin

**Affiliations:** 1 Mathematical Biosciences Institute, The Ohio State University, 231 W. 18th Avenue, Columbus, OH 43210, USA; 2 Department of Molecular Virology, Immunology, and Medical Genetics, Columbus, OH 43210, USA; 3 Human Cancer Genetics Program, Comprehensive Cancer Center, The Ohio State University, 420 W. 12th Avenue, Columbus, OH 43210, USA; 4 Department of Statistics, The Ohio State University, 1598 Neil Avenue, Columbus, OH 43210, USA; 5 Cleveland Clinic Genomic Medicine Institute, Cleveland Clinic Foundation, 9500 Euclid Avenue, Cleveland, OH 44195, USA

## Abstract

**Background:**

In order to recapitulate tumor progression pathways using epigenetic data, we developed novel clustering and pathway reconstruction algorithms, collectively referred to as heritable clustering. This approach generates a progression model of altered DNA methylation from tumor tissues diagnosed at different developmental stages. The samples act as surrogates for natural progression in breast cancer and allow the algorithm to uncover distinct epigenotypes that describe the molecular events underlying this process. Furthermore, our likelihood-based clustering algorithm has great flexibility, allowing for incomplete epigenotype or clinical phenotype data and also permitting dependencies among variables.

**Results:**

Using this heritable clustering approach, we analyzed methylation data obtained from 86 primary breast cancers to recapitulate pathways of breast tumor progression. Detailed annotation and interpretation are provided to the optimal pathway recapitulated. The result confirms the previous observation that aggressive tumors tend to exhibit higher levels of promoter hypermethylation.

**Conclusion:**

Our results indicate that the proposed heritable clustering algorithms are a useful tool for stratifying both methylation and clinical variables of breast cancer. The application to the breast tumor data illustrates that this approach can select meaningful progression models which may aid the interpretation of pathways having biological and clinical significance. Furthermore, the framework allows for other types of biological data, such as microarray gene expression or array CGH data, to be integrated.

## Background

Recapitulating pathways of tumor progression by tracing specific molecular lesions is necessary for understanding the disease and for developing novel drug targets and therapies. The idea of utilizing DNA methylation profiles to recapitulate tumor progression is even more enticing in that these epigenetic marks are stable and heritable in tumor genomes [1]. Specifically, this event occurs by the addition of a methyl group to a cytosine residue of a CpG dinucelotide [2]. It is recognized that in the normal genome, DNA methylation plays a role in mammalian development, imprinting, and X chromosome inactivation [3]. Recent advances further highlight a critical role of epigenetically mediated gene silencing in tumorigenesis [1]. Unmethylated CpG islands, located in the promoter regions of tumor suppressor/gatekeeper genes, become densely methylated during tumorigenesis [4-6]. Once the de novo methylation takes place, this new mark is maintained in subsequent cycles of cell replication by DNA methyltransfereases and other associated proteins, like polycomb repressors [7,8]. The consequence of these molecular events is a gradual accumulation of DNA methylation in an affected promoter CpG island. In addition, methylation-associated silencing of tumor suppressor genes can result in cells with a growth advantage. The number of hypermethylated genes tends to increase in more malignant cells, and clonal expansion of proliferating cells may generate specific tumor types marked by their unique epigenetic signatures [4,6]. This epigenetic event is inherently stable, and the silencing information is stored in the DNA methylation code of a tumor. Therefore, DNA methylation analysis can be retrospectively performed on clinical samples, allowing for studies of tumor progression history and for clinicopathological correlation. With the implementation of the state-of-the-art microarray technologies, it is now possible to obtain methylation signatures of multiple genes simultaneously and to classify tumors based on their global methylation patterns [9-12]. The idea of conducting a human epigenome project has recently been conceptualized [13] and is expected to facilitate our fundamental understanding of aberrant epigenetic mechanisms in cancer. In this study, we developed novel clustering and pathway reconstruction algorithms, collectively called heritable clustering, to evaluate a set of methylation microarray data previously generated in our laboratory [14]. Progressive accumulation of hypermethylated CpG islands was used to characterize breast tumor progression pathways. Utilizing these novel algorithms, we correlated specific methylation profiles with patient's clinical phenotypes and reconstructed the epigenetic history germane to tumorigenesis.

### Tumor progression pathways and recapitulation

**Figure 1:**
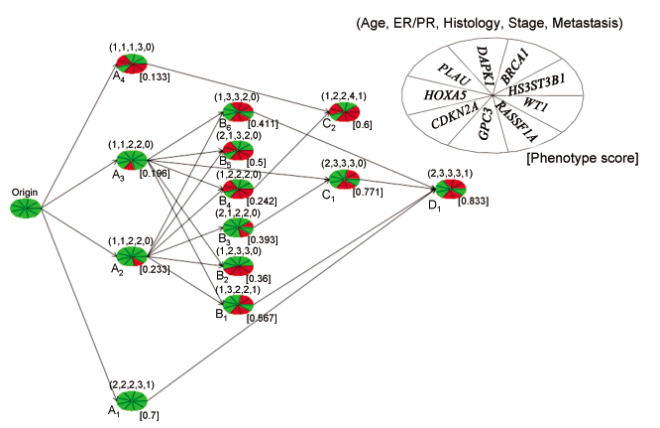
**Progression pathway network with *w *= 0.8, *ε *= 0.8 and the LH algorithm**. The methylation data analyzed here are from 86 primary breast cancers. A set of 9 gene promoter CpG islands is investigated. The methylation statuses of the genes in each node are represented in a color-coded pie chart, with red signifying hypermethylation while green denoting lack of differential methylation. There are five phenotype measurements for each tumor. They are: age (1, age > 55; 2, *age *≤ 55), ER/PR (1, +/+; 2, +/- or -/+; 3, -/-), histology (1, well-differentiated; 2, moderately-differentiated; 3, poorly-differentiated), clinical stage (1, 2, 3, or 4), and metastasis status (0, M0; 1, M1). The phenotype center for each phenotype is listed above each node in the order described above. The pathway network presented here conforms to strict heritability and tumor phenotype progression.

Abstractly, a tumor progression pathway is a directed graph with nodes corresponding to archetypal tumor stages and a directed edge denoting possible progression from one stage to another (see Figure 1). Tumor progression pathways are constructed based on the following characteristics: (1) most CpG islands are unmethylated in normal cells, (2) CpG island hypermethylation is heritable in tumor cells, and (3) multiple methylated loci are progressively accumulated during tumorigenesis. Based on these properties, we hypothesized that tumor cells have unique epigenetic signatures that are associated with specific cancer subtypes (phenotypic information). Specifically, we seek to construct patterns and relationships among hypermethylated genes that are progressively accumulated during tumorigenesis. As it is not possible for us to obtain tissues from the same patients at different stages of tumor progression over time, methylation data derived from tumors of different patients are used as surrogates for reconstructing tumor progression history. To accommodate the heritable nature of de novo methylation, a progression pathway ought to adhere to the notion that the hypermethylated loci acquired at each node are passed on to subsequent node(s). For two nodes *A *and *B*, *A *is an "ancestor" of *B *and *B *is a "progeny" of *A *if there exist nodes *V*_1_(= *A*),…, *V*_*n*_(= *B*) such that there is a directed edge from *V*_*i *_to *V*_*i*+1_, *i *= 1,… , *n *- 1 (*i.e*., a directed path from *A *to *B*). With this provided nomenclature, the progressive accumulation of hypermethylated loci is captured in the progression pathway by requiring that ancestor's methylated loci are subsets of their progeny's. Furthermore, the phenotypes of progeny nodes are hypothesized to be more aggressive than those of the ancestors'. Although existing clustering algorithms (e.g., hierarchical clustering or K-means) are available for clustering samples, no suitable method can be applied to give temporal directions of progression among different epigenetic clusters. In general, clustering algorithms (see [15] for a review and comparison of clustering methods most widely used for analyzing microarray data), including those that have been recently devised for progression modeling with genomic data (e.g., [16,17]), treat all observed events as belonging to terminal nodes. This is in contrast with the type of progression models that we wish to build to accommodate the hidden temporal structure so that all nodes, terminal or internal, contain the observed events. Such a challenge impedes us from adopting published clustering algorithms without major modifications. Therefore, we developed the heritable clustering algorithms to identify and organize clusters into a pathway and to recreate tumor progression pathways.

## Results

Three stages of heritable clustering are laid out in detail in Methods. We outline them here briefly before describing their application to a primary breast tumor dataset and the results. First, we determined the number of clusters, and second we assigned the tumor samples into clusters. Finally, the clusters were organized into a pathway structure to capture tumor progression. Other well-known clustering methods were also considered as alternatives. Except for the likelihood approach, which is based on probabilistic modeling, all the other methods considered here make use of a distance metric (see Methods).

### Data

Methylation analyses were initially performed on 93 intraductal carcinoma from unrelated patients, and their sample amplicons were deposited on the array [14]. The studied gene probes were hybridized to the array sequentially to generate composite methylation signatures [14]. A total of 10 genes were studied for their methylation status (0, unmethylated; 1, hypermethylated) in these tumor samples. These genes were chosen for analysis because of their known involvement in tumor suppression [18]. For a description of the methods used for generating the methylation data as well as assigning the discrete methylation values see [14]. Since gene *BRCA2 *is not methylated in any tumors, it is excluded from the final data analysis and model building. The remaining 9 genes used for pathway recapitulation are *GPC3*, *RASSF1A*, *WT1*, *PLAU*, *HOXA5*, *CDKN2A*, *HS3ST3B1*, *BRCA1*, and *DAPK1 *(for a detailed description, see Table 1). There are also five clinical phenotypes, and the categorical values of each phenotype are considered ordinal, with the lowest level to be adjusted to 0 for the heritable clustering analysis:

**Table 1:** Genes used in model construction.

Gene Symbol	Accession Number	GeneID	Gene Description	Function
*GPC3*	NM_004484	2719	glypican 3	Cell surface heparan sulfate proteoglycans; may play a role in the control of cell division and growth regulation
*RASSF1A*	NM_007182	11186	Ras association domain family 1	Protein similar to the RAS effector proteins; interact with DNA repair protein XPA; inhibit cyclin D1 accumulation thereby inducing cell cycle arrest
*WT1*	NM_000378	7490	Wilms tumor 1	A transcription factor; mutated in a small subset of patients with Wilm's tumours
*PLAU*	NM_002658	5328	plasminogen activator, urokinase	A serine protease involved in degradation of the extracellular matrix.
*HOXA5*	NM_019102	3202	homeobox	A DNA-binding transcription factor which may regulate gene expression, morphogenesis, and differentiation.
*CDKN2A*	NM_000077	1029	cyclin-dependent kniase inhibitor 2A	Functions as a stabilizer of the tumor suppressor protein p53.
*HS3ST3B1*	NM_006041	9953	heparan sulfate 3-O-sulfotransferase 3B1	An enzyme that possesses heparan sulfate glu-cosaminyl 3-O-sulfotransferase activity
*BRCA1*	NM_007294	672	breast cancer 1, early onset	A nuclear phosphoprotein that plays a role in maintaining genomic stability and acts as a tumor suppressor
*DAPK1*	NM_004938	1612	death-associated protein kinase 1	A positive mediator of gamma-interferon induced programmed cell death

• Yl = age (1: age > 55; 2: age ≤ 55),

• Y2 = Estrogen Receptor (ER)/Progesterone Receptor (PR) (1: +/+; 2: +/- or -/+; 3: -/-),

• Y3 = histology (1: well-differentiated; 2: moderately-differentiated; 3: poorly-differentiated),

• Y4 = clinical stage (1, 2, 3, or 4), and

• Y5 = metastasis status (1: no; 2: yes).

Of the 93 samples for which epigenotypes are available, seven have missing data on some of the phenotypic measurements. Therefore only the 86 samples that have complete data on both epigenotypes and phenotypes were used in the analyses presented in this section to facilitate comparisons among methods, although the likelihood method is amenable to the full set of 93 samples.

### Number of clusters

For the 86 samples with complete data, our model selection method was employed to find the optimal number of clusters and its associated parameter values for weight and similarity. Specifically, to apply our model selection procedure, we considered parameters *w *and *ε *in the range of 0.2 and 0.8, and 0.5 and 1, respectively, in increments of 0.1. We arranged the resulting values of the objective function *f*(*w*, *ε*) in ascending order. The result that optimizes our objective function has 13 clusters, with both the *w *and *ε *values being 0.8, as shown in Table 2. Also shown in the table are the next four best results according to the criterion.

**Table 2:** The top five clustering outcomes (ranked by the values of the objective function *f*) and the corresponding *w *and *ε *values.

Rank	*w*	*ε*	#Cluster(K)	Total Similarity (*T*_*S*_)	*f *(*w*, *ε*)
1	0.8	0.8	13	12.10	1.56
2	0.7	0.8	17	15.86	1.55
3	0.5	0.7	10	8.95	1.48
4	0.3	0.7	10	8.80	1.46
5	0.2	0.8	22	20.73	1.46

### Clustering analysis

We applied the two clustering algorithms detailed in Methods, SIM (similarity/distance based) and LH (likelihood based), to group the 86 tumor samples into 13 clusters. For the LH approach, the nine epigenotypes were treated as independent binomial variables, as was the age phenotype. However, since ER/PR status and histology (*ρ *= 0.46; *p *< 0.0001) were significantly positively correlated, these two variables were modeled jointly as follows:

*p*_0 _= *P*(|*Y*2 - *Y*3| = 0), *p*_1 _= *P*(|*Y*2 - *Y*3| = 1), and *p*_2 _= *P*(|*Y*2 - *Y*3| = 2) = 1 - *p*_0 _- *p*_1_. Similarly, the high positive correlation between clinical stage and metastasis (*ρ *= 0.62; *p *< = 0.0001) led us to the joint modeling of these two variables, which in essence was a binomial probability distribution with parameter *p *= *P*((*Y*4 ≤ 2 &*Y*5 = 1) or (*Y*4 ≥ 2 &*Y*5 = 2)).

In addition to these two novel clustering methods, we also analyzed the same set of data using three popular clustering methods in the literature, namely, K-means, PAM, and hierarchical clustering (H-clust), setting the number of clusters to 13. For these three popular algorithms and SIM, the distance measure was as described in Methods with *w *and *ε *set to correspond to the choice of the optimal number of clusters (Table 2), which uses both phenotypic and epigenotypic data. For all algorithms, the starting clustering assignments all use the by-product from the model selection step.

Three objective criteria, likelihood, silhouette, and entropy, were used to evaluate the outcomes of the various clustering algorithms. These criteria either try to measure the tightness of the samples within each cluster (likelihood and entropy) or the separation between clusters (silhouette). Our results in Table 3 show that LH outperformed the others under the likelihood and entropy criteria, with SIM being second in both cases. On the other hand, SIM came out a winner as far as the silhouette criterion is concerned. Both PAM and H-clust were not too far off from the optimal achieved by SIM in terms of silhouette, but they are not competitive under the other two criteria. The performance of K-means is also mixed. While it did a descent job evaluated under the entropy criterion, its likelihood and silhouette values are quite far from the optimal ones.

**Table 3:** Goodness-of-fit test for different cluster methods with different criteria.

Method	LH	SIM	K-means	PAM	H-clust
-log(LH)	70.05	85.05	97.15	107.73	97.88
Entropy	64.79	65.82	68.10	86.29	82.94
Silhouette	0.08	0.41	0.28	0.36	0.40

### Pathway recapitulation

Shown in Figure 1 is the recapitulated pathway built from the nodes derived from the LH algorithm, which performed the best for two of the three criteria evaluated. The red wedges in each pie (denoting a node) correspond to hypermethylated loci, while their green counterparts represent the loci that are not hypermethylated. The legend accompanying the figure annotate the organization of the nine loci. The numbers in the parentheses above each node are the phenotype centers arranged in the same order as that described in the Data subsection. The numbers in the brackets at the bottom right of each node is a node phenotype score defined by the phenotype center (see Methods). Finally, the pathway network built adheres to strict heritability.

### Interpretation

The progression pathway presented in Figure 1 depicts the outcome from PD using the methylation profiles of 9 promoter CpG islands found to be hypermethylated in primary breast tumors. These promoter CpG islands were selected because they are linked with known tumor-associated and/or tumor suppressor genes and their expression levels were shown previously to be perturbed by promoter hypermethylation. In this progression pathway, the proposed clustering method selects node centers that not only preserve strict heritability of promoter methylation but also uncover pathways with perfect progression in the 5 selected breast tumor phenotypes. This is a sound approach to construct the model as tumor phenotypes progress in a reasonably predictable manner (e.g., from small tumors to large tumors and from tumors that are contained within the primary site to tumors that have metastasized). This coupled with the cumulative nature of promoter methylation in genes whose expressions are known to be perturbed by methylation become the basis of our model. As such, we propose that tumors with more aggressive phenotypes should exhibit higher levels of methylation in this gene panel than the less aggressive tumors. A key utility of the reconstructed progression pathway is that it provides the opportunity to visualize the relationship between methylated gene promoters and the phenotype score. As readily apparent in Figure 1, this algorithm portrays complex and non-linear interplays between the methylation data and the phenotype scores.

#### Age phenotype

The first phenotype under consideration is the age of diagnosis. It is known that a young age of tumor onset generally correlates with a more aggressive disease. Often DNA methylation plays less of a role in tumorigenesis in this subset as evident in Nodes Al and B3. There are 47 patients older than 55 and 39 patients younger than 55. As such, an age of 55 is a reasonable cutoff to segregate pre-menopausal patients (thus patients with the more aggressive cancer, phenotype measurement = 2) from the post-menopausal patients (phenotype measurement = 1). This distinction is clearly illustrated by the phenotype summary of Node Al verses those of Nodes A2, and A3. All of these nodes are made up of tumors with little to no promoter methylation in the 9 studied genes, yet Nodes A2 and A3 had favorable phenotype scores of 0.233 and 0.196, respectively, while Node Al had a metastasis phenotype and a phenotype score of 0.7. This age effect is also evident when we examine all of the node phenotype score (an average score of 0.34 for old age tumor vs. 0.63 for young age tumor).

#### Hormone receptor status

ER/PR status is another phenotype that distinguishes early stage, less aggressive tumors from late stage, more aggressive tumors. Therefore, tumors expressing a measurable level of ER and PR (an assigned value of 1 or 2) should be clustered to the early and less aggressive nodes of the pathway while tumors without ER and PR expression should appear closer to the terminal nodes. Upon inspection of Figure 1, we noted the phenotype score for ER/PR = 1 or 2 is 0.37 and 0.63 for ER/PR = 3.

#### Tumor metastasis and histology

Another tumor phenotype that should follow stringent progression is tumor metastasis. A tumor that has shed a portion of its cells to distant sites such as lymph nodes represents a late stage, aggressive tumor. As such, tumors with a metastasis value of 1 should not appear in a node before tumors with no metastasis (metastasis value = 0). Tumor phenotypes relating to histology and tumor staging should progress similarly from a low grade or stage to a high grade or stage in the progression pathway. An intriguing observation is that nodes with a metastasis value of 1 have methylation events on average in 5 of the genes whereas nodes without metastasis has on average 3.6 methylation events. Node Al is an exception in that methylation events of these 9 genes are not involved in this archetypal tumor.

#### Promoter hypermethylation

Previous analysis on this data set showed that a large number of tumors have concurrent hypermethylation in the promoters of *GPC3 *and *RASSF1A *[12]. The progression pathway presented in Figure 1 shows that promoter methylation of these two genes is an early event in tumorigenesis as evident by the presence of hypermethylation in either *RASSF1A *or *GPC3 *or both in Nodes A2, A3, and A4. In the same vein, methylation of *WT1 *and *HS3ST3B *gene promoters seem to be a late event in that *WT1 *methylation occurs in 3 out of 4 terminal nodes (Nodes B2, B5, and C2) whereas *HS3ST3B *occurs in 2 out of 4 terminal nodes (Nodes C2 and D1). *BRCA1 *encodes a tumor suppressor gene that functions, in part, in maintaining genomic stability. In this progression pathway, *BRCA1 *methylation occurs exclusively in ER/PR negative tumors (Nodes B1, B6, C1, and D1). This is intriguing in that most *BRCA1 *mutant breast cancers are hormone receptor negative and here we showed that BRCA1 hypermethylation is also associated with hormone receptors negativity [19]. We also noticed that hypermethylation occurs concurrently in *PLAU *and *HOXA5 *as evident in Nodes A4, B4, B5, and C2. Both of these genes are known to be associated with apoptosis regulation in the mammary gland involution [20]. Having both of these gene promoters methylated would indeed confer survival advantage to the tumor clones. These types of relationships will not be at all apparent without breaking down the data according to clusters of common phenotype measurements.

## Discussion and conclusion

In this paper, we have developed novel methods for each of the three stages of the heritable clustering procedure. Although existing clustering methods are applicable to the second stage of the procedure, our proposed clustering algorithms, SIM and LH, outperformed their counterparts based on three objective evaluation criteria, for the data examined. Furthermore, our heritable clustering procedure seems to be able to capture the biological essence of tumor progression, as discussed below in general, and as elaborated in the above section for the breast tumor example in particular. Armed with these encouraging results from the breast tumor data, we plan to apply this framework to build progression models for genome-wide stroma and tumor methylation data that we are currently generating. However, in order for our proposed method to be applicable, judicious selection of relevant loci is an indispensable pre-processing step. In this regard, the resulting plausible and interpretable pathway model from our application to the breast tumor data owes largely to the set of loci that are known to be tumor-associated and/or tumor suppressor genes. Furthermore, since our focus of the current paper is on the particular breast tumor data, and our observations of the satisfactory performance of the heritable clustering methods are based on them, further assessment of the performance of heritable clustering for data from other tumor types is warranted in future applications.

The preliminary application of the heritable clustering algorithms to the breast tumor data demonstrates its effectiveness in identifying pathways with unambiguous epigenetic and phenotypic progression. The constructed pathway summarizes the epigenetic and phenotypic data in a way that corresponds to the current understanding of tumor progression. Further, the potential of methylation profiles to be used for characterizing tumor progression has been demonstrated. The resulting pathways from our tumor progression pathway recapitulation procedure depend on a number of factors including: 1) distance between tumors (epigenotype and phenotype); 2) balance between epigenotype and phenotype data; 3) similarities within clusters; and 4) heritability between nodes. The best results are those that reflect the underlying biological processes that lead to the formation of the primary tumors. Our heritable clustering method is designed based on the assumption that epigenetic changes are stably passed from progenitor to progeny cells [6]. Depending on what stage each tumor is diagnosed, some might have accumulated more epigenetic alternations than others as they have progressed more. In this paper, we capitalize on these epigenetic hallmarks to recapitulate breast tumor progression pathways utilizing CpG island hypermethylation data. In building the tumor progression pathway, the assumption is based on the heritable nature of CpG island hypermethylation passing from the parent node to its progeny nodes as tumor progresses. Therefore, the progeny nodes of tumor cells accumulate more hypermethylated gene promoters as they are further along in the progression pathway. The progeny tumor cells are likely to be more aggressive and have more proliferative advantages than the parental cells. Hence, we built a tumor progression model by linking the nodes or clusters based on strict heritability and their phenotype scores.

In practice, it is unlikely to recreate a linear temporal clinicopathological history of a cancer developing over time in a single patient as it is unethical to remove part of the tumor and allows a portion to grow for research purposes. To overcome this challenge common to all human genetic and epigenetic studies, we propose to view CpG island hypermethylation as "molecular relics" whereby one can trace how much each tumor has progressed by examining the overall methylation profile as such information is stably transmitted from parent cells to their progeny. The heritable clustering method developed in this paper is designed to uncover the different paths breast tumors can progress. Our results from the breast tumor application indicate that this approach is likely to select meaningful progression models and hopefully will assist in the interpretation of pathways having biological and clinical significance.

In this present application of the heritable clustering method, the epigenetic and clinical phenotypic values took on discrete values. However, the method can be extended to analyze other data types where the numerical values of the data are continuous. For instance, the method is well suited for modeling methylation data expressed as intensity ratios from two-color microarray experiments or transcription factor binding enrichment on gene promoters from ChIP-on-chip experiments. The framework would also allow for other types of biological data, such as microarray gene expression or array CGH data, to be integrated. Particular characteristics could also be employed in the selection of genes to be used directly or to be used collectively as a phenotype in the construction of tumor progression pathway. For example, 'CpG island methylator phenotype' (CIMP) is a distinct trait studied extensively in colorectal cancer. In colorectal cancer, a high frequency of Type C (cancer-associated methylation) loci was recently described by Weisenberger et al. [21]. The authors believe that they have arrived at a gene panel to classify CIMP comprised of unique underlying genotypes associated with microsatellite instability and BRAF mutation. The use of the methylation status of this panel of CIMP genes or the use of this panel collectively as a CIMP phenotype (by performing cluster analysis to group tumors into CIMP+ or CIMP-) to construct the tumor progression pathway recapitulation would add another dimension to the model. However, we wish to note that the value of using CIMP may be cancer-type dependent. For example, whereas CIMP is clearly valued for studying colorectal cancer, whether it should be used in building breast tumor progression pathway is debatable as much less is known about CIMP in breast cancer.

## Methods

The three stages of heritable clustering are laid out in detail here. First, we will describe an algorithm for determining the number of clusters. Then, we discuss two new procedures for clustering that are appropriate for our problem. Finally, we provide an algorithm that organizes the clusters into a pathway network to capture tumor progression epigenetically and phenotypically. Except for the likelihood approach, which is based on probabilistic modeling, all the other methods considered here make use of a distance metric. Thus, we describe our chosen distance, or the equivalent – similarity measure, next before we detail the three stages of heritable clustering.

### Similarity measures for epigenotypes and phenotypes

For our purposes, the data generated by methylation microarray are interpreted as categorical in nature (henceforth described as epigenotype) – 1: hypermethylated; 0: unmethylated. Methylation progression patterns among tumor samples are integral to the inheritance property of our model; hence, the capacity to capture such patterns is a requisite of any clustering method employed. Under these constraints, we choose to design our algorithm based on the concept of *ε*-similarity [22], which defines distance and similarity measures suitable for our analysis. Specifically, the Hamming distance [23] defines the distance between two binary vectors of equal length as the number of elements that have different bits. This distance measure is adopted for describing the distance between the epigenotypes of two tumor samples. For each tumor *t*, *t *= 1,… , *T*, let ***X***_*t *_= {*X*_*tg*_, *g *= 1,… , *G*} be the epigenotype vector at *G *loci. The epigenotype distance between two tumor samples *t*_*i *_and *t*_*j *_is then defined as

dg(i,j)=1G∑g=1G|Xtig−Xtjg|.
 MathType@MTEF@5@5@+=feaafiart1ev1aaatCvAUfKttLearuWrP9MDH5MBPbIqV92AaeXatLxBI9gBaebbnrfifHhDYfgasaacH8akY=wiFfYdH8Gipec8Eeeu0xXdbba9frFj0=OqFfea0dXdd9vqai=hGuQ8kuc9pgc9s8qqaq=dirpe0xb9q8qiLsFr0=vr0=vr0dc8meaabaqaciaacaGaaeqabaqabeGadaaakeaacqWGKbazdaWgaaWcbaGaem4zaCgabeaakiabcIcaOiabdMgaPjabcYcaSiabdQgaQjabcMcaPiabg2da9maalaaabaGaeGymaedabaGaem4raCeaamaaqahabaGaeiiFaWNaemiwaG1aaSbaaSqaaiabdsha0naaBaaameaacqWGPbqAaeqaaSGaem4zaCgabeaakiabgkHiTiabdIfaynaaBaaaleaacqWG0baDdaWgaaadbaGaemOAaOgabeaaliabdEgaNbqabaGccqGG8baFaSqaaiabdEgaNjabg2da9iabigdaXaqaaiabdEeahbqdcqGHris5aOGaeiOla4caaa@4F08@

Since most phenotype data (e.g., clinical stage, histological grade, or hormone receptor status) are categorical in nature, we assume that each tumor phenotype is a discrete ordinal, or can be ordered sensibly beginning from 0 to *K*_*p *_- 1, where *K*_*p *_is the number of the categories for phenotype *p*. Similar to the notation for epigenotypes, we use ***Y***_*t *_= {*Y*_*tp*_, *p *= 1,… , *P*} to denote the vector of phenotypes for tumor *t*. Then the phenotype distance between two tumors *t*_*i *_and *t*_*j *_is

dp(i,j)=1P∑p=1P|Ytip−Ytjp|Kp−1.
 MathType@MTEF@5@5@+=feaafiart1ev1aaatCvAUfKttLearuWrP9MDH5MBPbIqV92AaeXatLxBI9gBaebbnrfifHhDYfgasaacH8akY=wiFfYdH8Gipec8Eeeu0xXdbba9frFj0=OqFfea0dXdd9vqai=hGuQ8kuc9pgc9s8qqaq=dirpe0xb9q8qiLsFr0=vr0=vr0dc8meaabaqaciaacaGaaeqabaqabeGadaaakeaacqWGKbazdaWgaaWcbaGaemiCaahabeaakiabcIcaOiabdMgaPjabcYcaSiabdQgaQjabcMcaPiabg2da9maalaaabaGaeGymaedabaGaemiuaafaamaaqahabaWaaSaaaeaacqGG8baFcqWGzbqwdaWgaaWcbaGaemiDaq3aaSbaaWqaaiabdMgaPbqabaWccqWGWbaCaeqaaOGaeyOeI0IaemywaK1aaSbaaSqaaiabdsha0naaBaaameaacqWGQbGAaeqaaSGaemiCaahabeaakiabcYha8bqaaiabdUealnaaBaaaleaacqWGWbaCaeqaaOGaeyOeI0IaeGymaedaaaWcbaGaemiCaaNaeyypa0JaeGymaedabaGaemiuaafaniabggHiLdGccqGGUaGlaaa@5423@

Finally, the similarity measure between two tumors *t*_*i *_and *t*_*j *_is defined as

*S*(*t*_*i*_, *t*_*j*_) = 1 - (*w*·*d*_*p*_(*i*, *j*) + (1 - *w*)·*d*_*g*_(*i*, *j*)),

where 0 ≤ *w *≤ 1 is a weight parameter to balance the contributions from epigenotype and phenotype similarities. Two tumors, *t*_*i *_and *t*_*j*_, are said to be *ε*-similar if and only if *S*(*t*_*i*_, *t*_*j*_) ≥ *ε*, where 0 ≤ *ε *≤ 1 represents the level of similarity. In the proposed heritable clustering method, if two tumors are sufficiently similar, they will be clustered into the same group. The selection of an appropriate *ε *depends on the desired degree of similarity within a cluster. The lower the *ε *value, the less similarity (i.e. more variation) within each cluster is allowed. To balance the contributions from epigenotypes and phenotypes, and to guarantee a reasonable level of similarities among tumor samples within each cluster, we suggest considering the parameters *w *and *ε *in the following ranges: 0.2 ≤ *w *≤ 0.8 and 0.5 = *ε *≤ 1.

### Determination of number of clusters

For each combination of weight and similarity (*w*, *ε*) (e.g., 0.2 ≤ *w *≤ 0.8,0.5 ≤ *ε *≤ 1), we use the following steps to determine the number of clusters.

1. Begin with two tumors {*t*_*i*_, *t*_*j*_} that maximize the similarity measure *S*(*t*_*i*_, *t*_*j*_). If the maximal value of S is less than *ε*, assign each tumor into separate clusters and stop. Otherwise let *C*_1 _= {*t*_*i*_, *t*_*j*_} and go to the next step.

2. Suppose there exist *K *clusters *C*_1_,… , *C*_*k*_. Let *n*_*k *_be the number of tumors in *C*_*k *_and *t*_*ki *_be the *i*-th tumor to be added to *C*_*k*_, *k *= 1,… , *K*, and *i *= 1,… , *n*_*k*_. Let *t *∈ *T *be a tumor sample that has not yet been assigned to any of the clusters. The similarity score between *t *and each of the existing cluster is defined as:

S(t,Ck)=∑i=1nkS(t,tki)/nk.
 MathType@MTEF@5@5@+=feaafiart1ev1aaatCvAUfKttLearuWrP9MDH5MBPbIqV92AaeXatLxBI9gBaebbnrfifHhDYfgasaacH8akY=wiFfYdH8Gipec8Eeeu0xXdbba9frFj0=OqFfea0dXdd9vqai=hGuQ8kuc9pgc9s8qqaq=dirpe0xb9q8qiLsFr0=vr0=vr0dc8meaabaqaciaacaGaaeqabaqabeGadaaakeaacqWGtbWucqGGOaakcqWG0baDcqGGSaalcqWGdbWqdaWgaaWcbaGaem4AaSgabeaakiabcMcaPiabg2da9maaqahabaGaem4uamLaeiikaGIaemiDaqNaeiilaWIaemiDaq3aaSbaaSqaaiabdUgaRjabdMgaPbqabaGccqGGPaqkcqGGVaWlcqWGUbGBdaWgaaWcbaGaem4AaSgabeaaaeaacqWGPbqAcqGH9aqpcqaIXaqmaeaacqWGUbGBdaWgaaadbaGaem4AaSgabeaaa0GaeyyeIuoakiabc6caUaaa@4C58@

Let (t*, k*) = argmax{*S*(*t*, C_*k*_), *t *∈ *T*, *k *= 1,… , *K*}. If *S*(t*, *C*_k*_) ≥ *ε*, then *C*_k* _= *C*_k* _∪ {t*}; otherwise create a new cluster *C*_*K*+1 _= {t*}.

3. Repeat step 2 until all tumor samples are assigned to clusters. Then calculate the total similarity score

TS(w,ε)=∑k=1K(w,ε)ASk,
 MathType@MTEF@5@5@+=feaafiart1ev1aaatCvAUfKttLearuWrP9MDH5MBPbIqV92AaeXatLxBI9gBaebbnrfifHhDYfgasaacH8akY=wiFfYdH8Gipec8Eeeu0xXdbba9frFj0=OqFfea0dXdd9vqai=hGuQ8kuc9pgc9s8qqaq=dirpe0xb9q8qiLsFr0=vr0=vr0dc8meaabaqaciaacaGaaeqabaqabeGadaaakeaacqWGubavdaWgaaWcbaGaem4uamfabeaakiabcIcaOiabdEha3jabcYcaSGGaciab=v7aLjabcMcaPiabg2da9maaqahabaGaemyqaeKaem4uam1aaSbaaSqaaiabdUgaRbqabaaabaGaem4AaSMaeyypa0JaeGymaedabaGaem4saSKaeiikaGIaem4DaCNaeiilaWIae8xTduMaeiykaKcaniabggHiLdGccqGGSaalaaa@4704@

where ASk=∑i=1nk∑j=1nkS(tki,tkj)/nk2
 MathType@MTEF@5@5@+=feaafiart1ev1aaatCvAUfKttLearuWrP9MDH5MBPbIqV92AaeXatLxBI9gBaebbnrfifHhDYfgasaacH8akY=wiFfYdH8Gipec8Eeeu0xXdbba9frFj0=OqFfea0dXdd9vqai=hGuQ8kuc9pgc9s8qqaq=dirpe0xb9q8qiLsFr0=vr0=vr0dc8meaabaqaciaacaGaaeqabaqabeGadaaakeaacqWGbbqqcqWGtbWudaWgaaWcbaGaem4AaSgabeaakiabg2da9maaqadabaWaaabmaeaacqWGtbWucqGGOaakcqWG0baDdaWgaaWcbaGaem4AaSMaemyAaKgabeaakiabcYcaSiabdsha0naaBaaaleaacqWGRbWAcqWGQbGAaeqaaOGaeiykaKIaei4la8IaemOBa42aa0baaSqaaiabdUgaRbqaaiabikdaYaaaaeaacqWGQbGAcqGH9aqpcqaIXaqmaeaacqWGUbGBdaWgaaadbaGaem4AaSgabeaaa0GaeyyeIuoaaSqaaiabdMgaPjabg2da9iabigdaXaqaaiabd6gaUnaaBaaameaacqWGRbWAaeqaaaqdcqGHris5aaaa@534E@ is the average similarity in cluster *k *and *K*(*w*, *ε*) is the corresponding number of clusters with parameters *w *and *ε*.

What remains is to find the optimal cluster number *K *and its corresponding parameter pair (*w*, *ε*). In general, if the number of clusters *K *is large, then *T*_*S *_is also large, and consequently log(*T*_*S*_) is also large. This leads us to propose a model selection criterion following the formulation of Akaike's Information Criterion (AIC) [24]. The main idea is to maximize total similarity subject to a penalty term for over stratification. Specifically, we seek (*K*, *w*, *ε*) that satisfies

(K,w,ε)=arg⁡max⁡{f(w,ε)=log⁡(TS(w,ε))−K(w,ε)P+G;  0.2≤w≤0.8, 0.5≤ε≤1},
 MathType@MTEF@5@5@+=feaafiart1ev1aaatCvAUfKttLearuWrP9MDH5MBPbIqV92AaeXatLxBI9gBaebbnrfifHhDYfgasaacH8akY=wiFfYdH8Gipec8Eeeu0xXdbba9frFj0=OqFfea0dXdd9vqai=hGuQ8kuc9pgc9s8qqaq=dirpe0xb9q8qiLsFr0=vr0=vr0dc8meaabaqaciaacaGaaeqabaqabeGadaaakeaacqGGOaakcqWGlbWscqGGSaalcqWG3bWDcqGGSaaliiGacqWF1oqzcqGGPaqkcqGH9aqpcyGGHbqycqGGYbGCcqGGNbWzcyGGTbqBcqGGHbqycqGG4baEcqGG7bWEcqWGMbGzcqGGOaakcqWG3bWDcqGGSaalcqWF1oqzcqGGPaqkcqGH9aqpcyGGSbaBcqGGVbWBcqGGNbWzcqGGOaakcqWGubavdaWgaaWcbaGaem4uamfabeaakiabcIcaOiabdEha3jabcYcaSiab=v7aLjabcMcaPiabcMcaPiabgkHiTmaalaaabaGaem4saSKaeiikaGIaem4DaCNaeiilaWIae8xTduMaeiykaKcabaGaemiuaaLaey4kaSIaem4raCeaaiabcUda7iabbccaGiabbccaGiabicdaWiabc6caUiabikdaYiabgsMiJkabdEha3jabgsMiJkabicdaWiabc6caUiabiIda4iabcYcaSiabbccaGiabicdaWiabc6caUiabiwda1iabgsMiJkab=v7aLjabgsMiJkabigdaXiabc2ha9jabcYcaSaaa@79EA@

where *P *and *G *denote the number of phenotypes and epigenotypes, respectively, as defined before. Note that the second term in the objective function *f *is to penalize over estimation of the number of clusters. It is designed to balance the number of clusters and total similarity, as in AIC.

A different clustering algorithm other than the one described above may be used to determine the number of clusters for each *w *and *ε*. However, existing algorithms cannot easily accommodate the requirement of *ε*-similarity, which is what prompted us to devise the above algorithm.

### Clustering samples

We then turn to clustering algorithms to group samples into *K *clusters, where *K *is the optimal number of clusters determined from the previous stage. Here we describe two novel algorithms, one based on distance (similarity) and the other based on likelihood. Both methods are iterative procedures like that of k-means, and therefore it is worth noting that the first stage of heritable clustering also produces clusters as a by-product, which can be conveniently used as initial clusters here. For the three existing distance-based algorithms that we also consider, K-means, PAM, and hierarchical clustering, the distance measure used is the same as that for SIM, to be described, which uses both methylation status and phenotypes. We note that this distance measure differs from what usually used in these popular algorithms (e.g., Euclidean distance or correlation based) as we are dealing with discrete data and want to take both epigenotype and phenotype information into consideration. However, the proposed method is equally applicable to continuous data or a combination of continuous/discrete data from an operational point of view, albeit with a different distance measure suitable for the data type(s).

#### Distance-based similarity approach

This can be regarded as a hybrid clustering algorithm that combines the essence of K-means and Silhouette (principal criterion in PAM) to balance within-cluster similarity and between-cluster distinctiveness. Let *a*(*t*) denote the average similarity between tumor *t *and all the other tumors in cluster *C*_k* _to which *t *belongs. For any of the other clusters *C*_*k*_, *k *≠ k*, let *S*(*t*, *C*_*k*_) be the average similarity of *t *to all samples in *C*_*k*_. We denote by *b*(*t*) = max_*k *≠ k* _*S*(*t*, *C*_*k*_) the similarity between *t *and its nearest "neighbor" cluster. If *ab*(*t*) = *a*(*t*) - *b*(*t*) ≥ 0, we say that *t *is correctly assigned to its current cluster, *C*_k*_, otherwise, it is a candidate for switching its cluster membership.

Algorithm: SIM

1. Calculate *ab*(*t*) for each *t *and let *abmin *= min_*t *_*ab*(*t*).

2. If *abmin *< 0, move the corresponding *t *to its "neighbor" cluster.

3. Repeat 1–2 until *abmin *≥ 0.

#### Likelihood-based method

Unlike the SIM algorithm or most other existing algorithms in the literature, the likelihood-based method proposed here does not depend on any measure of distance. This leads to greater flexibility, in that the approach can deal with both discrete and continuous data, missing observations for some of the variables, and dependencies among the variables. The idea is similar, in spirit, to that reported in Cai et al. [25] for SAGE data. It also shares the commonality of a parametric clustering formulation with Siegmund et al. [26] for analyzing methylation data, although the two address two completely different problems. We assume that the epigenotype vectors (***X***_*t*_) for tumor *t *follows a common parametric family of distributions with its own parameter vector ***θ***_*tG*_. Analogously, we use ***θ***_*tp *_to denote the parameter vector for the distribution of the clinical phenotype vector ***Y***_*t*_. That is,

***X***_*t *_= {*X*_*t*1_, *X*_*t*2_,… , *X*_*tG*_}~*P*(. | *θ*_*tG*_),

***Y***_*t *_= {*Y*_*t*1_, Y_*t*2_,… , *Y*_*tP*_}~*P*(.| *θ*_*tP*_).

Thus, ***X***_*t *_and ***Y***_*t *_are jointly distributed as

(***X***_*t*_, ***Y***_*t*_) ~ *P*(.|*θ*_*t *_= (*θ*_*tG*_, *θ*_*tP*_)).

If ***X***_*t *_and ***Y***_*t *_are assumed to be independent, then

*P*(***X***_*t*_, ***Y***_*t *_| *θ*_*tG*_, *θ*_*tP*_) = *P*(***X***_*t *_| *θ*_*tG*_)*P*(***Y***_*t*_| *θ*_*tP*_).

The goal is to group tumors with similar epigenotypes and phenotypes according to their parameter vectors. That is, we assume that tumors within a cluster (*C*_*k*_) share the common distributional parameter vector θk={θGk,θPk}
 MathType@MTEF@5@5@+=feaafiart1ev1aaatCvAUfKttLearuWrP9MDH5MBPbIqV92AaeXatLxBI9gBaebbnrfifHhDYfgasaacH8akY=wiFfYdH8Gipec8Eeeu0xXdbba9frFj0=OqFfea0dXdd9vqai=hGuQ8kuc9pgc9s8qqaq=dirpe0xb9q8qiLsFr0=vr0=vr0dc8meaabaqaciaacaGaaeqabaqabeGadaaakeaaiiWacqWF4oqCdaahaaWcbeqaaiabdUgaRbaakiabg2da9iabcUha7jab=H7aXnaaDaaaleaacqWGhbWraeaacqWGRbWAaaGccqGGSaalcqWF4oqCdaqhaaWcbaGaemiuaafabaGaem4AaSgaaOGaeiyFa0haaa@3DB4@, which represents the cluster profile. Let *I*_*k*_(*t*) = 1 if tumor *t *is in cluster *C*_*k*_, otherwise it is 0, in the current iteration. Then, the joint likelihood is

L(θGk,θPk,k=1,⋯,K|Xt,Yt,t=1,⋯,T)=∏k=1K{∏t=1T[P(Xt,Yt|θGk,θPk)]Ik(t)},
 MathType@MTEF@5@5@+=feaafiart1ev1aaatCvAUfKttLearuWrP9MDH5MBPbIqV92AaeXatLxBI9gBaebbnrfifHhDYfgasaacH8akY=wiFfYdH8Gipec8Eeeu0xXdbba9frFj0=OqFfea0dXdd9vqai=hGuQ8kuc9pgc9s8qqaq=dirpe0xb9q8qiLsFr0=vr0=vr0dc8meaabaqaciaacaGaaeqabaqabeGadaaakeaacqWGmbatcqGGOaakiiWacqWF4oqCdaqhaaWcbaGaem4raCeabaGaem4AaSgaaOGaeiilaWIae8hUde3aa0baaSqaaiabdcfaqbqaaiabdUgaRbaakiabcYcaSiabdUgaRjabg2da9iabigdaXiabcYcaSiabl+UimjabcYcaSiabdUealjabcYha8Hqadiab+HfaynaaBaaaleaacqWG0baDaeqaaOGaeiilaWIae4xwaK1aaSbaaSqaaiabdsha0bqabaGccqGGSaalcqWG0baDcqGH9aqpcqaIXaqmcqGGSaalcqWIVlctcqGGSaalcqWGubavcqGGPaqkcqGH9aqpdaqeWbqaamaacmqabaWaaebCaeaadaWadaqaaiabdcfaqjabcIcaOiab+HfaynaaBaaaleaacqWG0baDaeqaaOGaeiilaWIae4xwaK1aaSbaaSqaaiabdsha0bqabaGccqGG8baFcqWF4oqCdaqhaaWcbaGaem4raCeabaGaem4AaSgaaOGaeiilaWIae8hUde3aa0baaSqaaiabdcfaqbqaaiabdUgaRbaakiabcMcaPaGaay5waiaaw2faamaaCaaaleqabaGaemysaK0aaSbaaWqaaiabdUgaRbqabaWccqGGOaakcqWG0baDcqGGPaqkaaaabaGaemiDaqNaeyypa0JaeGymaedabaGaemivaqfaniabg+GivdaakiaawUhacaGL9baaaSqaaiabdUgaRjabg2da9iabigdaXaqaaiabdUealbqdcqGHpis1aOGaeiilaWcaaa@81AA@

where *K *is the number of clusters, and *T *is the number of tumor samples. Suppose that θ^Gk
 MathType@MTEF@5@5@+=feaafiart1ev1aaatCvAUfKttLearuWrP9MDH5MBPbIqV92AaeXatLxBI9gBaebbnrfifHhDYfgasaacH8akY=wiFfYdH8Gipec8Eeeu0xXdbba9frFj0=OqFfea0dXdd9vqai=hGuQ8kuc9pgc9s8qqaq=dirpe0xb9q8qiLsFr0=vr0=vr0dc8meaabaqaciaacaGaaeqabaqabeGadaaakeaaiiWacuWF4oqCgaqcamaaDaaaleaacqWGhbWraeaacqWGRbWAaaaaaa@311D@ and θ^Pk
 MathType@MTEF@5@5@+=feaafiart1ev1aaatCvAUfKttLearuWrP9MDH5MBPbIqV92AaeXatLxBI9gBaebbnrfifHhDYfgasaacH8akY=wiFfYdH8Gipec8Eeeu0xXdbba9frFj0=OqFfea0dXdd9vqai=hGuQ8kuc9pgc9s8qqaq=dirpe0xb9q8qiLsFr0=vr0=vr0dc8meaabaqaciaacaGaaeqabaqabeGadaaakeaaiiWacuWF4oqCgaqcamaaDaaaleaacqWGqbauaeaacqWGRbWAaaaaaa@312F@ are the maximum likelihood estimate of θGk
 MathType@MTEF@5@5@+=feaafiart1ev1aaatCvAUfKttLearuWrP9MDH5MBPbIqV92AaeXatLxBI9gBaebbnrfifHhDYfgasaacH8akY=wiFfYdH8Gipec8Eeeu0xXdbba9frFj0=OqFfea0dXdd9vqai=hGuQ8kuc9pgc9s8qqaq=dirpe0xb9q8qiLsFr0=vr0=vr0dc8meaabaqaciaacaGaaeqabaqabeGadaaakeaaiiWacqWF4oqCdaqhaaWcbaGaem4raCeabaGaem4AaSgaaaaa@310D@ and θGk
 MathType@MTEF@5@5@+=feaafiart1ev1aaatCvAUfKttLearuWrP9MDH5MBPbIqV92AaeXatLxBI9gBaebbnrfifHhDYfgasaacH8akY=wiFfYdH8Gipec8Eeeu0xXdbba9frFj0=OqFfea0dXdd9vqai=hGuQ8kuc9pgc9s8qqaq=dirpe0xb9q8qiLsFr0=vr0=vr0dc8meaabaqaciaacaGaaeqabaqabeGadaaakeaaiiWacqWF4oqCdaqhaaWcbaGaem4raCeabaGaem4AaSgaaaaa@310D@, respectively, *k *= 1,… , *K*, then it is natural to evaluate how well a particular tumor sample fits into the assigned cluster by computing

k* = argmin_*k*_{- log *P*(***X***_*t*_, ***Y***_*t *_| θ^Gk
 MathType@MTEF@5@5@+=feaafiart1ev1aaatCvAUfKttLearuWrP9MDH5MBPbIqV92AaeXatLxBI9gBaebbnrfifHhDYfgasaacH8akY=wiFfYdH8Gipec8Eeeu0xXdbba9frFj0=OqFfea0dXdd9vqai=hGuQ8kuc9pgc9s8qqaq=dirpe0xb9q8qiLsFr0=vr0=vr0dc8meaabaqaciaacaGaaeqabaqabeGadaaakeaaiiWacuWF4oqCgaqcamaaDaaaleaacqWGhbWraeaacqWGRbWAaaaaaa@311D@, θ^Pk
 MathType@MTEF@5@5@+=feaafiart1ev1aaatCvAUfKttLearuWrP9MDH5MBPbIqV92AaeXatLxBI9gBaebbnrfifHhDYfgasaacH8akY=wiFfYdH8Gipec8Eeeu0xXdbba9frFj0=OqFfea0dXdd9vqai=hGuQ8kuc9pgc9s8qqaq=dirpe0xb9q8qiLsFr0=vr0=vr0dc8meaabaqaciaacaGaaeqabaqabeGadaaakeaaiiWacuWF4oqCgaqcamaaDaaaleaacqWGqbauaeaacqWGRbWAaaaaaa@312F@); *k *= 1,… , *K*}.

If *C*_k* _is not the same as its currently assigned cluster, then tumor *t *is a candidate for switching cluster membership. This basic idea may lead to various clustering algorithms, including the one below used for our primary breast tumor data.

The above formulation of the likelihood clustering approach provides a general setting in which dependencies among epigenotypes (e.g., hypermethylated promoter regions binded to the same transcription factor) and phenotypes (e.g, tumor grade and metastasis status) can be easily incorporated. In the breast tumor example, we have discrete epigenotypes (hypermethylated or not) and phenotypes (ordinal), therefore binomial and multinomial are the natural choice of parametric families for the distributions of the variables. However, the framework can be flexibly adapted to any other type of data, such as continuous measurements of methylation intensities. Finally, the approach can make use of tumor samples that have missing data on some of the variables; the contribution to the corresponding likelihood from such a sample will be set to unity by convention.

Algorithm: LH

1. For each tumor *t *∈ *C*_k*_, calculate *L*_*t*_(k*) = -log *P*(***X***_*t*_, ***Y***_*t*_|θ^k∗
 MathType@MTEF@5@5@+=feaafiart1ev1aaatCvAUfKttLearuWrP9MDH5MBPbIqV92AaeXatLxBI9gBaebbnrfifHhDYfgasaacH8akY=wiFfYdH8Gipec8Eeeu0xXdbba9frFj0=OqFfea0dXdd9vqai=hGuQ8kuc9pgc9s8qqaq=dirpe0xb9q8qiLsFr0=vr0=vr0dc8meaabaqaciaacaGaaeqabaqabeGadaaakeaaiiWacuWF4oqCgaqcamaaCaaaleqabaGaem4AaS2aaWbaaWqabeaacqGHxiIkaaaaaaaa@3123@) and *L*(*k *≠ k*) = min_*k*≠k*_{-log *P*(***X***_*t*_, ***Y***_*t*_|θ^k
 MathType@MTEF@5@5@+=feaafiart1ev1aaatCvAUfKttLearuWrP9MDH5MBPbIqV92AaeXatLxBI9gBaebbnrfifHhDYfgasaacH8akY=wiFfYdH8Gipec8Eeeu0xXdbba9frFj0=OqFfea0dXdd9vqai=hGuQ8kuc9pgc9s8qqaq=dirpe0xb9q8qiLsFr0=vr0=vr0dc8meaabaqaciaacaGaaeqabaqabeGadaaakeaaiiWacuWF4oqCgaqcamaaCaaaleqabaGaem4AaSgaaaaa@3006@)}.

2. If max_*t *_{*L*_*t*_(k*)/*L*_*t*_(*k *≠ k*)}*> *1, move the corresponding *t *to cluster Ck^
 MathType@MTEF@5@5@+=feaafiart1ev1aaatCvAUfKttLearuWrP9MDH5MBPbIqV92AaeXatLxBI9gBaebbnrfifHhDYfgasaacH8akY=wiFfYdH8Gipec8Eeeu0xXdbba9frFj0=OqFfea0dXdd9vqai=hGuQ8kuc9pgc9s8qqaq=dirpe0xb9q8qiLsFr0=vr0=vr0dc8meaabaqaciaacaGaaeqabaqabeGadaaakeaacqWGdbWqdaWgaaWcbaGafm4AaSMbaKaaaeqaaaaa@2F56@ where k^
 MathType@MTEF@5@5@+=feaafiart1ev1aaatCvAUfKttLearuWrP9MDH5MBPbIqV92AaeXatLxBI9gBaebbnrfifHhDYfgasaacH8akY=wiFfYdH8Gipec8Eeeu0xXdbba9frFj0=OqFfea0dXdd9vqai=hGuQ8kuc9pgc9s8qqaq=dirpe0xb9q8qiLsFr0=vr0=vr0dc8meaabaqaciaacaGaaeqabaqabeGadaaakeaacuWGRbWAgaqcaaaa@2E1B@ = argmin_*k*≠k*_{-log *P*(***X***_*t*_, ***Y***_*t*_|θ^k
 MathType@MTEF@5@5@+=feaafiart1ev1aaatCvAUfKttLearuWrP9MDH5MBPbIqV92AaeXatLxBI9gBaebbnrfifHhDYfgasaacH8akY=wiFfYdH8Gipec8Eeeu0xXdbba9frFj0=OqFfea0dXdd9vqai=hGuQ8kuc9pgc9s8qqaq=dirpe0xb9q8qiLsFr0=vr0=vr0dc8meaabaqaciaacaGaaeqabaqabeGadaaakeaaiiWacuWF4oqCgaqcamaaCaaaleqabaGaem4AaSgaaaaa@3006@)}.

3. Repeat 1–2 until max_*t *_≤ 1.

### Building progression pathway

In the final stage of heritable clustering, clusters generated from the previous stage will be assembled into a pathway structure to represent the pathway of tumorigenesis. We first describe the concepts of cluster centers and scores, which are essential for our pathway discovery (PD) algorithm.

The clusters as previously described become the nodes of the tumor progression model. In order to derive pathways between nodes, a vector representative of the epigenotype and phenotype signatures of the tumors within a given node needs to be defined. Node centers and scores (both epigenotypic and phenotypic) derived from each cluster are used to define such a vector and is referred to as the node label. The epigenotype center of a cluster is determined by the epigenotype status common to the majority of tumors in the cluster. Let *V*_*kg *_denote the set of epigenotype statuses at locus *g *over all tumors in cluster *C*_*k*_, and *P*(*V*_*kg*_) be the number of 1s in *V*_*kg*_. Then the *epigenetic node center *(*ENC*) for locus *g *in cluster *C*_*k *_is defined as:

GCkg={1,if P(Vkg)≥card{Vkg}/2;0,otherwise;
 MathType@MTEF@5@5@+=feaafiart1ev1aaatCvAUfKttLearuWrP9MDH5MBPbIqV92AaeXatLxBI9gBaebbnrfifHhDYfgasaacH8akY=wiFfYdH8Gipec8Eeeu0xXdbba9frFj0=OqFfea0dXdd9vqai=hGuQ8kuc9pgc9s8qqaq=dirpe0xb9q8qiLsFr0=vr0=vr0dc8meaabaqaciaacaGaaeqabaqabeGadaaakeaacqWGhbWrcqWGdbWqdaWgaaWcbaGaem4AaSMaem4zaCgabeaakiabg2da9maaceqabaqbaeaabiGaaaqaaiabigdaXiabcYcaSaqaaiabbMgaPjabbAgaMjabbccaGiabdcfaqjabcIcaOiabdAfawnaaBaaaleaacqWGRbWAcqWGNbWzaeqaaOGaeiykaKIaeyyzImRaee4yamMaeeyyaeMaeeOCaiNaeeizaqMaei4EaSNaemOvay1aaSbaaSqaaiabdUgaRjabdEgaNbqabaGccqGG9bqFcqGGVaWlcqaIYaGmcqGG7aWoaeaacqaIWaamcqGGSaalaeaacqqGVbWBcqqG0baDcqqGObaAcqqGLbqzcqqGYbGCcqqG3bWDcqqGPbqAcqqGZbWCcqqGLbqzcqGG7aWoaaaacaGL7baaaaa@6079@

where card{*V*_*kg*_} is the cardinality of the set *V*_*kg*_. The epigenotype score, or degree, of the node *k *is then defined based on the calculated node centers as follows:

GSk=1G∑g=1GGCkg,
 MathType@MTEF@5@5@+=feaafiart1ev1aaatCvAUfKttLearuWrP9MDH5MBPbIqV92AaeXatLxBI9gBaebbnrfifHhDYfgasaacH8akY=wiFfYdH8Gipec8Eeeu0xXdbba9frFj0=OqFfea0dXdd9vqai=hGuQ8kuc9pgc9s8qqaq=dirpe0xb9q8qiLsFr0=vr0=vr0dc8meaabaqaciaacaGaaeqabaqabeGadaaakeaacqWGhbWrcqWGtbWudaWgaaWcbaGaem4AaSgabeaakiabg2da9maalaaabaGaeGymaedabaGaem4raCeaamaaqahabaGaem4raCKaem4qam0aaSbaaSqaaiabdUgaRjabdEgaNbqabaaabaGaem4zaCMaeyypa0JaeGymaedabaGaem4raCeaniabggHiLdGccqGGSaalaaa@4031@

*i.e*., the proportion of 1s in the set of ENCs for the node. The epigenotype score of a node is interpreted as measuring the extent of methylation of the tumors within the cluster.

With the definition of epigenotype centers and scores, it is now possible to define heritability of a progeny *C*_*j *_from a parental node *C*_*i *_:

H(Ci,Cj)=card{g|GCig=GCjg=1,g=1,...,G}/GGSi.
 MathType@MTEF@5@5@+=feaafiart1ev1aaatCvAUfKttLearuWrP9MDH5MBPbIqV92AaeXatLxBI9gBaebbnrfifHhDYfgasaacH8akY=wiFfYdH8Gipec8Eeeu0xXdbba9frFj0=OqFfea0dXdd9vqai=hGuQ8kuc9pgc9s8qqaq=dirpe0xb9q8qiLsFr0=vr0=vr0dc8meaabaqaciaacaGaaeqabaqabeGadaaakeaacqWGibascqGGOaakcqWGdbWqdaWgaaWcbaGaemyAaKgabeaakiabcYcaSiabdoeadnaaBaaaleaacqWGQbGAaeqaaOGaeiykaKIaeyypa0ZaaSaaaeaacqqGJbWycqqGHbqycqqGYbGCcqqGKbazcqGG7bWEcqWGNbWzcqGG8baFcqWGhbWrcqWGdbWqdaWgaaWcbaGaemyAaKMaem4zaCgabeaakiabg2da9iabdEeahjabdoeadnaaBaaaleaacqWGQbGAcqWGNbWzaeqaaOGaeyypa0JaeGymaeJaeiilaWIaem4zaCMaeyypa0JaeGymaeJaeiilaWIaeiOla4IaeiOla4IaeiOla4IaeiilaWIaem4raCKaeiyFa0Naei4la8Iaem4raCeabaGaem4raCKaem4uam1aaSbaaSqaaiabdMgaPbqabaaaaOGaeiOla4caaa@5F58@

The value of *H*(*C*_*i*_, *C*_*j*_), which is between 0 and 1 and meaningful only if *GS*_*i *_≤ *GS*_*j*_, is the degree of heritability. Strict inheritance is defined when *H *= 1. Under this condition, all hypermethylated loci in a parental node are inherited by its progeny nodes. Note that the heritability is defined on the loci methylation signature of the ENC and not the methylation signature of the tumors that comprise the node. Such a definition of heritability is faithful to the recapitulation nature of the method. In an analogous manner, phenotype centers and scores are used to capture the clinical progression in tumorigenesis. The center of a phenotype in a cluster is taken to be the weighted average of the phenotype values of the samples in the cluster rounded to the nearest integer. Let *n*_*p *_be the number of categories for phenotype *p *and *c*_*ki *_= card{*Y*_*tp *_= *i*| *t *∈ *k*} be the count of category *i *in cluster *C*_*k*_, *i *= 1,… , *n*_*p*_. Then the phenotypic node center of phenotype *p *in cluster *C*_*k *_is

PCkp=⌊PCkp(nr)+0.5⌋=⌊∑i=1npi cki∑i=1npcki+0.5⌋,
 MathType@MTEF@5@5@+=feaafiart1ev1aaatCvAUfKttLearuWrP9MDH5MBPbIqV92AaeXatLxBI9gBaebbnrfifHhDYfgasaacH8akY=wiFfYdH8Gipec8Eeeu0xXdbba9frFj0=OqFfea0dXdd9vqai=hGuQ8kuc9pgc9s8qqaq=dirpe0xb9q8qiLsFr0=vr0=vr0dc8meaabaqaciaacaGaaeqabaqabeGadaaakeaacqWGqbaucqWGdbWqdaWgaaWcbaGaem4AaSMaemiCaahabeaakiabg2da9maagmaabaGaemiuaaLaem4qam0aa0baaSqaaiabdUgaRjabdchaWbqaaiabcIcaOiabd6gaUjabdkhaYjabcMcaPaaakiabgUcaRiabicdaWiabc6caUiabiwda1aGaayj84laawUp+aiabg2da9maagmaabaWaaSaaaeaadaaeWaqaaiabdMgaPjabbccaGiabdogaJnaaBaaaleaacqWGRbWAcqWGPbqAaeqaaaqaaiabdMgaPjabg2da9iabigdaXaqaaiabd6gaUnaaBaaameaacqWGWbaCaeqaaaqdcqGHris5aaGcbaWaaabmaeaacqWGJbWydaWgaaWcbaGaem4AaSMaemyAaKgabeaaaeaacqWGPbqAcqGH9aqpcqaIXaqmaeaacqWGUbGBdaWgaaadbaGaemiCaahabeaaa0GaeyyeIuoaaaGccqGHRaWkcqaIWaamcqGGUaGlcqaI1aqnaiaawcp+caGL7JpacqGGSaalaaa@6B47@

where ⌊ ⌋ is the floor of the value being bracketed.

The phenotype score for cluster *C*_*k *_is then calculated as

PSk=1P∑p=1PPCkp(nr)KP−1,
 MathType@MTEF@5@5@+=feaafiart1ev1aaatCvAUfKttLearuWrP9MDH5MBPbIqV92AaeXatLxBI9gBaebbnrfifHhDYfgasaacH8akY=wiFfYdH8Gipec8Eeeu0xXdbba9frFj0=OqFfea0dXdd9vqai=hGuQ8kuc9pgc9s8qqaq=dirpe0xb9q8qiLsFr0=vr0=vr0dc8meaabaqaciaacaGaaeqabaqabeGadaaakeaacqWGqbaucqWGtbWudaWgaaWcbaGaem4AaSgabeaakiabg2da9maalaaabaGaeGymaedabaGaemiuaafaamaaqahabaWaaSaaaeaacqWGqbaucqWGdbWqdaqhaaWcbaGaem4AaSMaemiCaahabaGaeiikaGIaemOBa4MaemOCaiNaeiykaKcaaaGcbaGaem4saS0aaSbaaSqaaiabdcfaqbqabaGccqGHsislcqaIXaqmaaaaleaacqWGWbaCcqGH9aqpcqaIXaqmaeaacqWGqbaua0GaeyyeIuoakiabcYcaSaaa@49A2@

where *K*_*p*_, as defined before, is the number of categories for phenotype *p*. This score can be interpreted as measuring the average phenotypic value of the tumors in the cluster, with a larger score being indicative of more advanced tumors. Analogous to the concept of epigenotype heritability, we assume that phenotypic scores follow a temporal order. That is, a node with a small score represents a tumor that occurred temporally before a tumor represented by a node with a larger score. Our PD algorithm is built to capture this chronological characteristic.

Algorithm: PD

1. Sort nodes in ascending order according to their epigenotypic scores with ties determined by their phenotypic scores. In the unlikely case that at least two nodes have the same epigenotypic and phenotypic scores, their ordering is determined randomly. Assume, with possible relabeling, that the set of ordered nodes is *C *= {1, 2, … , *K*}. Set node 1 as the initial node in the pathway network.

2. Suppose *C*^*N *^is the set of nodes already used to construct the pathway. The ordering of *C *determined in the previous step is inherited by *C*^*N*^. Let C* = *C*^*N*^, and also note that C* will be reset at each iteration in Step 3. Add the node *k *= min{*C*\*C*^*N*^} to the pathway (i.e., to *C*^*N *^but not to C*) and finding all possible directed paths to it from other nodes already in the pathway in Step 3.

3. While C* ≠ ∅:

Let *j *= max{C*}. If (a) *H*(*j*, *k*) ≥ *h*, and (b) for each phenotype *p*, *PC*_*jp *_≤ *PC*_*kp*_, then *k *is added as a downstream node to node *j*; then the node *j *and all nodes upstream of it (*i.e*., nodes *i *such that there exists a directed path from *i *to *j*) are removed from the set C*. Otherwise, only *j *is removed from the set C*.

4. Repeat Steps 2 and 3 until all nodes have been added to the pathway.

5. If there does not exist a node that is upstream of all other nodes, then a pseudo-origin is created by defining a node *C*_0 _with both phenotype and epigenotype score of zero. All nodes in *C*^*N *^without upstream nodes are added as downstream adjacent nodes of *C*_0_.

In step 1 of the PD algorithm, different ordering of the tied nodes will not lead to altered pathways. In fact, step 1 is needed only for designing an efficient algorithm. One may work with the nodes in any order, but then one would also need to check whether a new node to be added is a upstream node of a node already in the pathway (in addition to checking whether it is a downstream node). In this way, regardless of the ordering, the same pathway will result.

## Authors' contributions

ZW and SL developed the methodology with inputs from all other authors. ZW and DP carried out the data analyses and preparation of the tables and the figure. All authors contributed to the drafting of the manuscript. All authors read and approved the final manuscript.
